# Tetrahydrobiopterin prevents chronic ischemia-related lower urinary tract dysfunction through the maintenance of nitric oxide bioavailability

**DOI:** 10.1038/s41598-020-76948-9

**Published:** 2020-11-16

**Authors:** Hidenori Akaihata, Junya Hata, Ryo Tanji, Ruriko Honda-Takinami, Kanako Matsuoka, Yuichi Sato, Masao Kataoka, Soichiro Ogawa, Yoshiyuki Kojima

**Affiliations:** Department of Urology, Fukushima Medical University of School of Medicine, Fukushima, 960-1295 Japan

**Keywords:** Medicinal chemistry, Pharmacology, Cardiovascular biology

## Abstract

This study aimed to investigate the influence of chronic ischemia on nitric oxide biosynthesis in the bladder and the effect of administering tetrahydrobiopterin (BH4), a cofactor for endothelial nitric oxide synthase (eNOS), on chronic ischemia-related lower urinary tract dysfunction (LUTD). This study divided male Sprague–Dawley rats into Control, chronic bladder ischemia (CBI) and CBI with oral BH4 supplementation (CBI/BH4) groups. In the CBI group, bladder capacity and bladder muscle strip contractility were significantly lower, and arterial wall was significantly thicker than in Controls. Significant improvements were seen in bladder capacity, muscle strip contractility and arterial wall thickening in the CBI/BH4 group as compared with the CBI group. Western blot analysis of bladder showed expressions of eNOS (*p* = 0.043), HIF-1α (*p* < 0.01) and dihydrofolate reductase (DHFR) (*p* < 0.01), which could regenerate BH4, were significantly higher in the CBI group than in Controls. In the CBI/BH4 group, HIF-1α (*p* = 0.012) and DHFR expressions (*p* = 0.018) were significantly decreased compared with the CBI group. Our results suggest that chronic ischemia increases eNOS and DHFR in the bladder to prevent atherosclerosis progression. However, DHFR could not synthesize sufficient BH4 relative to the increased eNOS, resulting in LUTD. BH4 supplementation protects lower urinary tract function by promoting eNOS activity.

## Introduction

Lower urinary tract symptoms (LUTS), such as urgency, are associated with lower urinary tract dysfunction (LUTD) and are well known to increase with age in both sexes and to greatly affect quality of life (QOL)^1^. One cause of LUTD in both men and women is chronic bladder ischemia (CBI), which is induced by pelvic arterial occlusion, including by atherosclerosis^2,3^. Animal models of CBI have shown bladder hyperactivity, defined as a significantly increased frequency of micturition^4–8^. In male LUTS patients, severe atherosclerosis significantly decreased maximum flow rate and voided volume^9^. Clarification of the mechanisms underlying reductions in blood flow to pelvic structures is important for understanding the causes of LUTD.

The key mediator of vascular homeostasis is nitric oxide (NO), which plays important roles in regulating various functions in the cardiovascular system, such as inhibition of leukocyte-endothelial adhesion and proliferation and platelet aggregation^10^. NO bioavailability is reduced throughout the progression of atherosclerosis^11^. This results from both reduced synthesis and increased consumption of NO by reactive oxygen species (ROS). With progression of atherosclerosis, endothelial NO synthase (eNOS) may become enzymatically uncoupled and produce ROS rather than NO, resulting in vascular oxidative stress and endothelial dysfunction^12,13^. This switch between NO and ROS generation is determined by the availability of tetrahydrobiopterin (BH4)^14,15^. BH4 is well known as a cofactor for the activities of phenylalanine, tyrosine, and tryptophan hydroxylases during neurotransmitter synthesis, and oral administration of BH4 improves blood phenylalanine levels in BH4-responsive phenylketonuria patients. Recently, BH4 was also identified as a crucial cofactor in NO biosynthesis^16,17^. Deficiency of BH4 led to the uncoupling of NOS, with a shift toward ROS generation^18^. BH4 is synthesized by either the de novo or salvage pathway. The de novo pathway starts with guanosine triphosphate (GTP), which is metabolized to BH4 by GTP cyclohydrolase I (GCH-1) and sepiapterin (SP) reductase (SR). The rate-limiting enzyme in the de novo pathway is GCH-1. The salvage pathway converts SP and dihydrobiopterin (BH2) to BH4 by SR and dihydrofolate reductase (DHFR)^19^. However, whether chronic ischemia affects NO and BH4 synthase in the bladder has not been established. On the other hand, administration of BH4 promotes the generation of NO and other antioxidant effects, which improve endothelium-dependent vasomotion in several pathological states, including arterial hypertension, coronary heart disease and ischemic reperfusion^20–22^. Whether administration of BH4 influences LUTD in arterial occlusion-induced CBI is also unclear.

We therefore used a rat model of CBI^6–8^ to investigate the influence of chronic ischemia on NO and BH4 synthase in the bladder and the effects of BH4 on chronic ischemia-related LUTD.

## Results

### Body weight and bladder wet weight did not differ among the three groups

Sixteen-week-old male Sprague–Dawley rats were divided into Control, CBI and CBI with BH4 supplementation (CBI/BH4) groups. The CBI group underwent balloon endothelial injury of bilateral iliac arteries, and were fed a 2% cholesterol diet for 8 weeks after the procedure to induce CBI. The CBI/BH4 group received the same procedure and diet as the CBI group. In addition, rats in the CBI/BH4 group were orally administered BH4 at 10 mg/kg/day for 8 weeks. Among the three groups, body weight and bladder wet weight did not differ significantly (Table 1).

**Table 1:** Body weight and bladder wet weight at 8 weeks in Control, CBI and CBI/BH4 group rats.

	Control (n = 10)	CBI (n = 10)	CBI/BH4 (n = 10)
Body weight (g)	531.0 ± 25.6	536.0 ± 34.1	516.4 ± 18.6
Bladder wet weight (g)	0.24 ± 0.04	0.24 ± 0.04	0.26 ± 0.07

### Cystometric parameters were protected in the CBI/BH4 group

**Figure 1:**
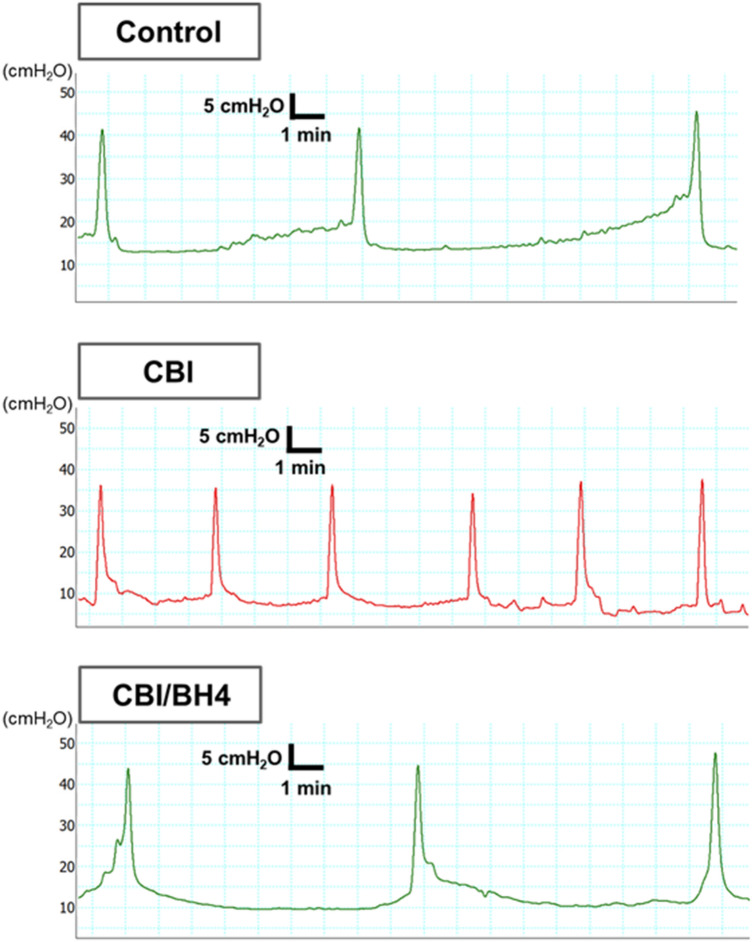
Typical cystometrogram recordings in Control, CBI and CBI/BH4 group rats. Micturition interval is significantly shorter in the CBI group than in the other groups. Scale bars represent 5 min and 5 cm H_2_O.

Cystometry was performed in conscious, freely moving rats. Bladder capacity (Bcap) and mean voided volume (VV) were significantly less and micturition interval (MI) was significantly shorter in the CBI group than in the Control group (*p* < 0.01 each). Bcap and VV were significantly higher, and MI was significantly longer in the CBI/BH4 group than in the CBI group (Bcap, *p* = 0.014; VV, *p* < 0.01; MI, *p* = 0.014). No differences in residual volume, basal pressure, threshold pressure, maximum pressure or bladder compliance were evident among the three groups (Fig. 1, Table 2).

**Table 2:** Cystometric parameters at 8 weeks in Control, CBI and CBI/BH4 group rats.

	Control (n = 10)	CBI (n = 10)	CBI/BH4 (n = 10)
Micturition interval (min)	12.44 ± 3.32	7.58 ± 2.45*	11.43 ± 2.60**
Bladder capacity (mL)	2.07 ± 0.55	1.26 ± 0.41*	1.91 ± 0.43**
Voided volume (mL)	1.99 ± 0.58	1.20 ± 0.40*	2.00 ± 0.51**
Residual volume (mL)	0.11 ± 0.09	0.09 ± 0.11	0.03 ± 0.05
Baseline pressure (cmH_2_O)	12.88 ± 1.57	11.72 ± 4.63	11.22 ± 4.14
Threshold pressure (cmH_2_O)	25.47 ± 4.90	20.20 ± 5.64	20.63 ± 6.63
Maximum pressure (cmH_2_O)	42.82 ± 7.33	40.13 ± 9.86	43.33 ± 8.58
Bladder compliance (mL/cmH_2_O)	0.18 ± 0.07	0.17 ± 0.08	0.23 ± 0.10

Values represent mean ± standard deviation.

Micturition interval: time period between two maximum voiding pressures.

Bladder capacity: infused volume/number of micturitions.

Voided volume: volume after a micturition − volume before a micturition, as registered in a urine collection cup.

Residual volume: bladder capacity − voided volume.

Baseline pressure: minimum pressure between two micturitions.

Threshold pressure: intravesical pressure immediately before micturition.

Maximum pressure: Maximum bladder pressure during a micturition cycle.

Bladder compliance: bladder capacity/(threshold pressure − baseline pressure).

*p < 0.05: versus Control, **p < 0.05: versus CBI.

### Muscle strip contractility was not impaired in the CBI/BH4 group

**Figure 2:**
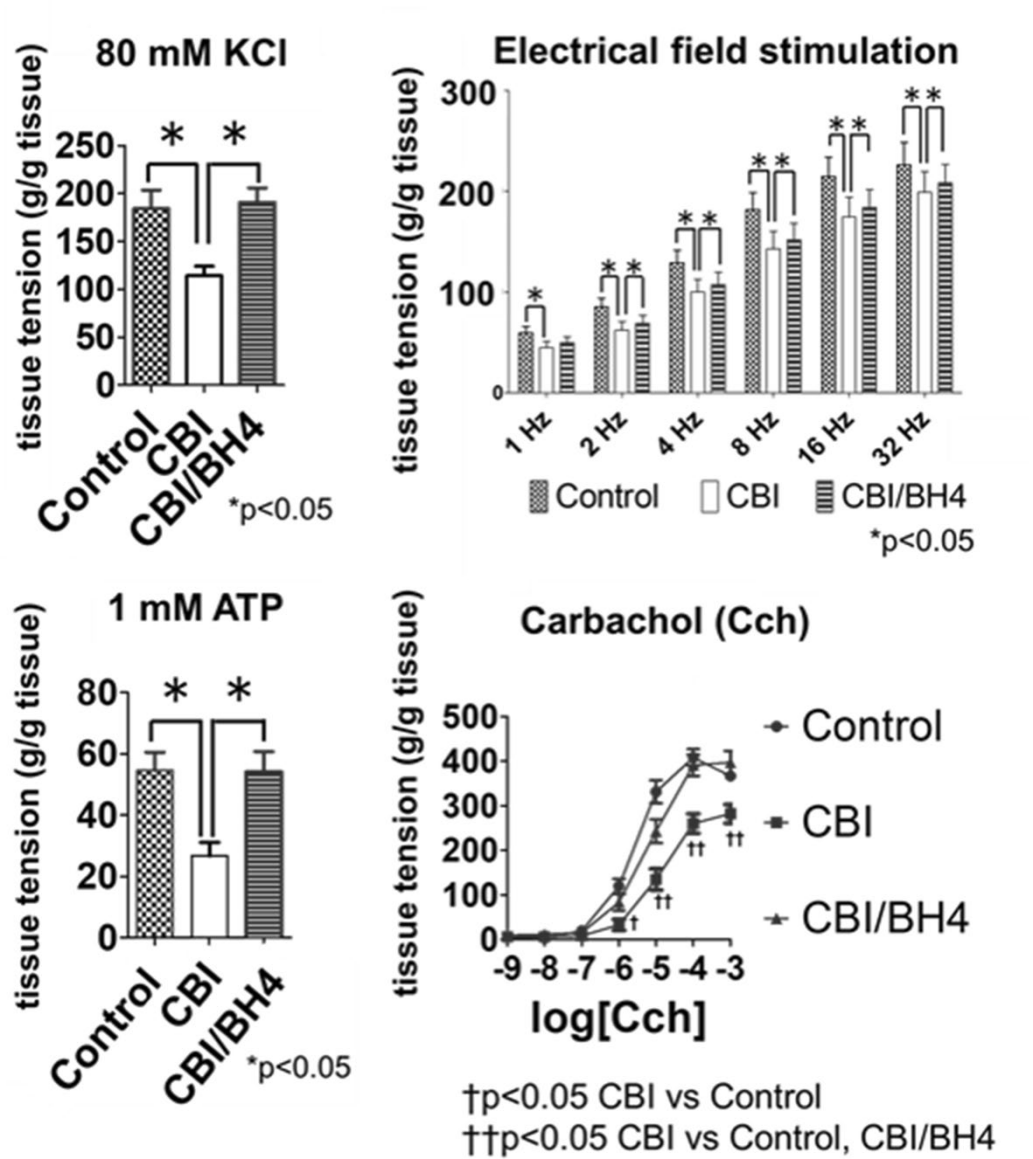
Contractile responses to KCl, electrical field stimulation, ATP and carbachol in control, CBI and CBI/BH4 rats (n = 10 each). Mean contractile responses to KCl, 1 mM ATP, electrical field stimulation and carbachol at concentrations from 1 μM to 1 mM were significantly weaker in the CBI group than in the other groups. Values represent mean ± standard error of the mean (SEM). Asterisk indicates statistical significance at the *p* < 0.05 level. ^†^*p* < 0.05 in CBI versus Control groups. ^††^*p* < 0.05 in CBI versus Control and CBI/BH4 groups.

Each muscle strip from bladders was suspended in an organ bath (25 ml) containing Krebs’ solution. Mean contractile responses to KCl, 1 mM adenosine triphosphate (ATP), EFS and carbachol at concentrations from 1 μM to 1 mM were significantly weaker in the CBI group than in the Control group. The CBI/BH4 group showed significantly stronger contractile responses to KCl, 1 mM ATP, EFS and carbachol at concentrations from 10 μM to 1 mM as compared with the CBI group (Fig. 2). The 50% effective concentration (EC_50_) was calculated from carbachol dose–response curves. EC_50_s of carbachol were 1.9 ± 1.2 μM in the Control group, 16.2 ± 1.2 μM in the CBI group, and 8.7 ± 1.2 μM in the CBI/BH4 Group (Values were EC50 ± standard error of the mean).

### Arterial occlusion and bladder fibrosis did not occur in the CBI/BH4 group and eNOS-positive cells on the endothelium were reduced in the Control group

**Figure 3:**
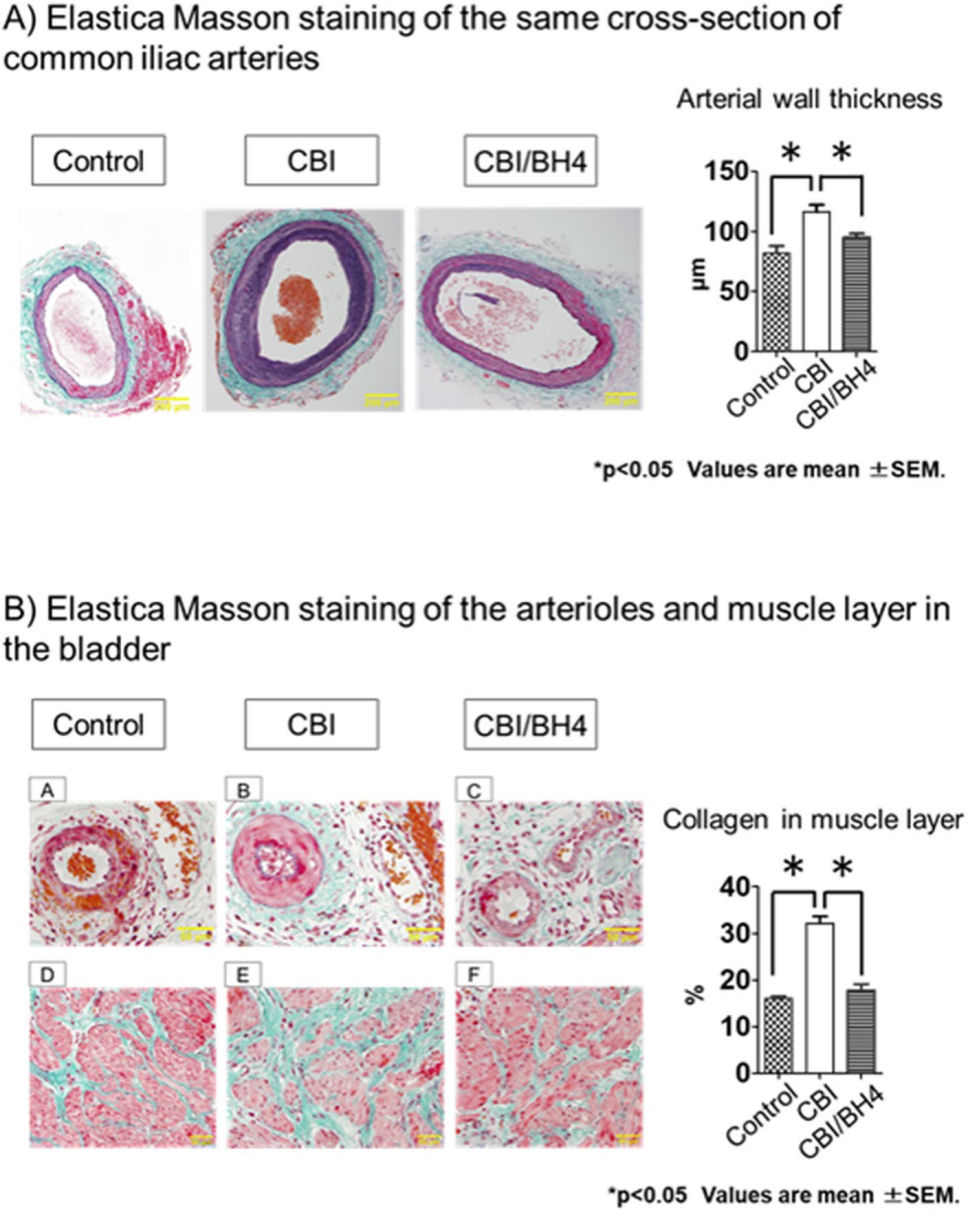

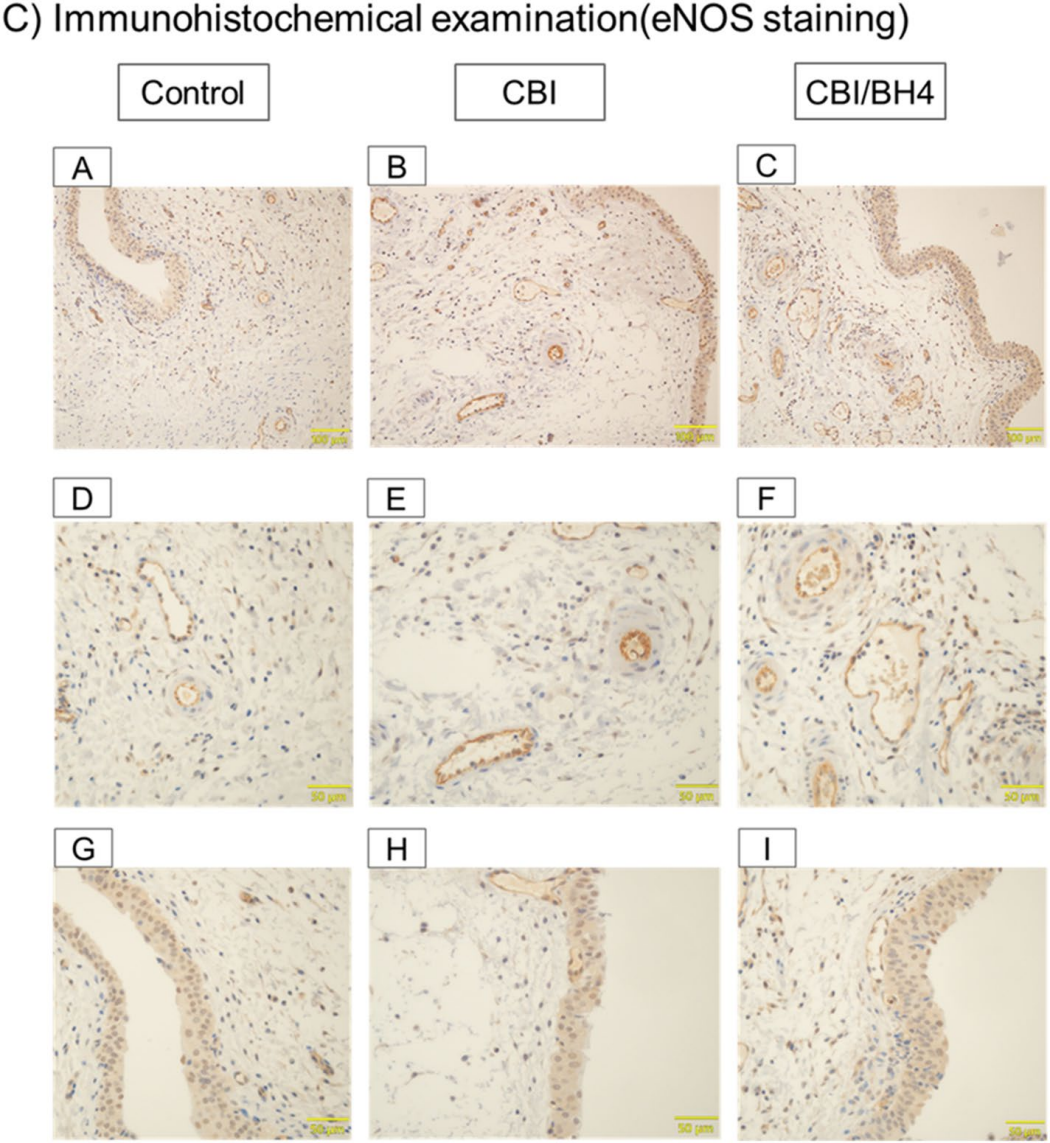
(**A**) Elastica Masson staining of the same cross-sections of common iliac artery from rats in the Control (10 rats, 20 vessels), CBI (10 rats, 20 vessels) and CBI/BH4 (10 rats, 20 vessels) groups (100 × magnification; scale bars, 200 μm). Bar graphs show average wall thickness of the common iliac artery in Control, CBI and CBI/BH4 groups. Elastica Masson staining of cross-sections of common iliac artery from the CBI group demonstrates mean arterial wall thickness with neointimal formation is significantly greater in the CBI group than in the other groups. Values represent mean ± SEM. *p < 0.05. (**B**) Elastica Masson staining of arterioles (upper panel) and muscle layer (lower panel) in bladder from Control (A,D), CBI (B,E) and CBI/BH4 (C,F) groups (400 × magnification; scale bars, 50 μm). Bar graphs show the percentage of collagen in the muscle layer of the Control, CBI and CBI/BH4 groups. Elastica Masson staining of bladder arterioles shows obvious arterial wall thickening compared with other groups. In the CBI group, the percentage of collagen in the muscle layer is significantly increased as compared with other groups. Values represent mean ± SEM. *p < 0.05. (**C**) Immunohistochemical examinations of bladder from Control (A,D,G), CBI (B,E,H) and CBI/BH4 (C,F,I) groups (upper panel: 200 × magnification; scale bars, 100 μm. middle and lower panel: 400 × magnification; scale bars 50 μm). Middle panels show vessels and lower panels show urothelium. In the Control group, eNOS-positive cells on the vascular endothelium were markedly reduced as compared with other groups.

Elastica Masson (EM) staining showed that mean arterial wall thickness of the common iliac arteries was significantly greater in the CBI group than in the Control group (*p* < 0.01). In the CBI/BH4 group, mean arterial wall thickness of common iliac arteries was significantly decreased as compared with the CBI group (*p* = 0.014). In addition, the CBI group revealed obvious severe arterial occlusive disease of bladder arterioles compared with other groups. EM staining of bladder tissue demonstrated that the percentage of collagen in the muscle layer of the CBI group was significantly increased compared with the Control group (*p* < 0.01). EM staining also showed a significantly decreased percentage of collagen in the muscle layer of bladder in the CBI/BH4 group as compared with the CBI group (*p* < 0.01). With immunohistochemical staining, eNOS-positive cells were located mostly on vascular endothelium and urothelium in the bladder. In the Control group, eNOS-positive cells on the vascular endothelium were markedly reduced as compared with other groups (Fig. 3).

### Expressions of DHFR and eNOS were increased in the CBI group

**Figure 4:**
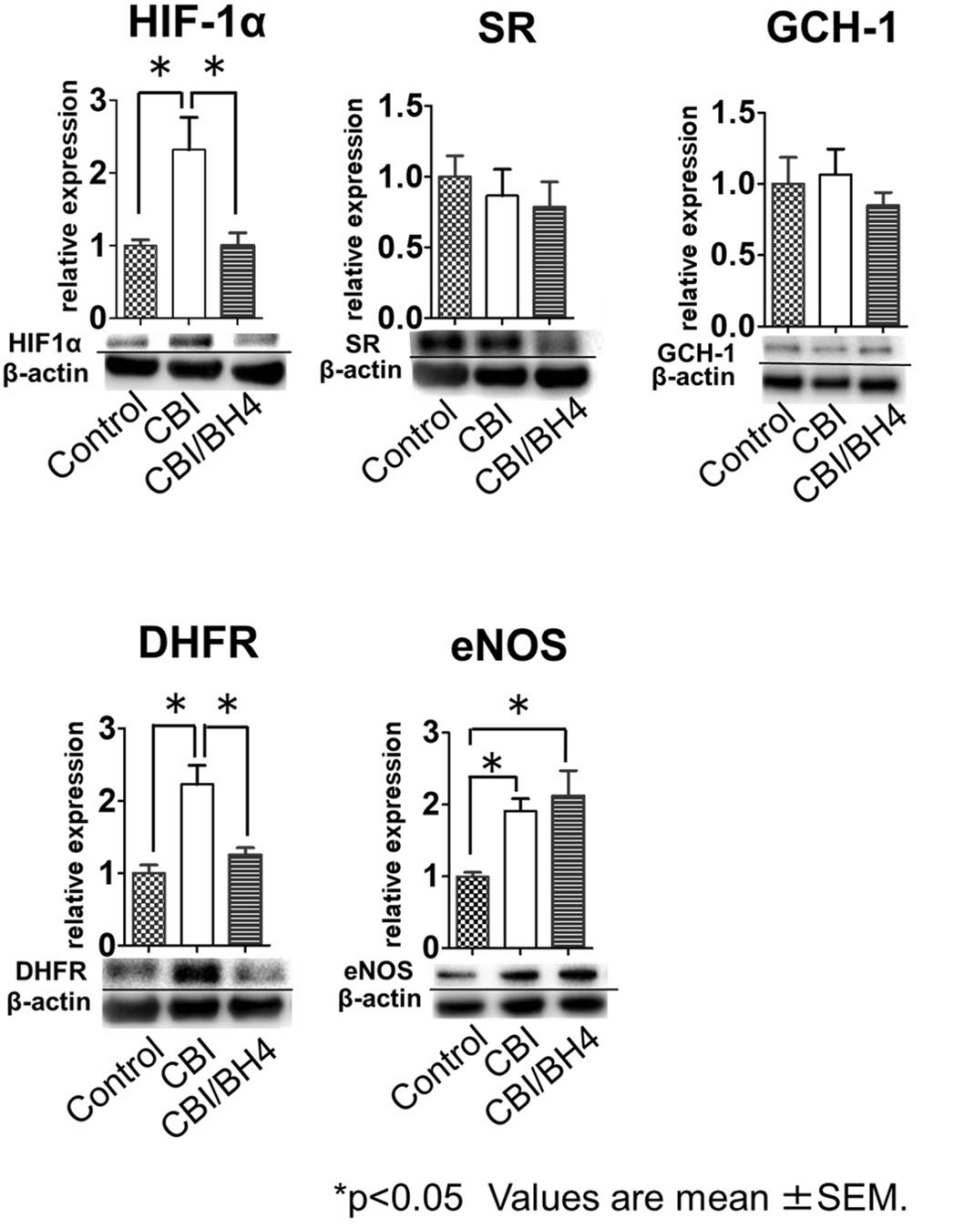
Expression of signaling targets at the protein level in the bladder. Groupings of gels/blots were cropped from different parts of the same gel. We washed the membranes by WB Stripping Solution Strong to allow two uses. With the first wash, anti-HIF-1α, anti-GCH-1, anti-SR, anti-DHFR or anti-eNOS was used. With the second wash, anti-β actin was used every time. Expression of HIF-1α, an oxidative stress marker, is significantly increased in the CBI group as compared with the other groups. Expression of eNOS is significantly higher in the CBI group and CBI/BH4 group than in the Control group. In the CBI group, expression of DHFR, which can regenerate BH4 from BH2 via the salvage pathway of BH4 synthesis, is significantly increased compared with the other groups. No significant differences in SR or GCH-1 expression are evident among the three groups. Each bar represents the mean ± SEM of 6–9 determinations, each from a different bladder. *p < 0.05. HIF-1α: hypoxia-inducible factor 1; GCH-1: guanosine triphosphate cyclohydrolase 1; SR: sepiapterin reductase; DHFR: dihydrofolate reductase; eNOS: endothelial nitric oxide synthase.

Western blotting using bladder tissue showed that expressions of HIF-1α (*p* < 0.01) and DHFR (*p* < 0.01), which can regenerate BH4 from BH2 in the salvage pathway of BH4 synthesis, were significantly higher in the CBI group than in the Control group. Expression of eNOS was also significantly increased in the CBI group as compared with the Control group (*p* = 0.043). In the CBI/BH4 group, expressions of HIF-1α (*p* = 0.012) and DHFR (*p* = 0.018) were significantly decreased as compared with the CBI group. No significant differences in eNOS expression were evident between the CBI and CBI/BH4 groups. On the other hand, eNOS expression was significantly higher in the CBI/BH4 group than in the Control group (*p* < 0.01). No significant difference in SR and GCH-1 expression was found among the three groups (Fig. 4).

## Discussion

In this study, the CBI group showed arterial wall thickening in both bladder arterioles and common iliac arteries. Arterial balloon endothelial injury with a high-cholesterol diet has been demonstrated to induce arterial occlusion in downstream bladder microvessel, as well as at sites of iliac artery injury in rats and rabbits^4–6^. Potent vasoconstrictive substances such as thromboxane A2 and 5-hydroxytryptamine are suggested to be produced in damaged endothelial sites, causing downstream microvascular spasm and remodeling^23,24^. We confirmed previous observations that pelvic arterial occlusion causes ischemia/reperfusion injury and bladder hyperactivity, defined as shortened MI, decreased Bcap and VV, without affecting maximum pressure or residual volume in the CBI group^6,8,25^. In our organ bath studies, EC_50_s from carbachol dose–response curves might indicate that pelvic arterial occlusion affected muscarinic receptor function in the bladder and that BH4 administrations protected the muscarinic receptors.

Our findings also demonstrated overexpression of eNOS in the bladder of the CBI rat model. While eNOS plays a key role in preventing endothelial dysfunction by synthesizing NO^10^, eNOS overexpression paradoxically shows detrimental effects in the setting of atherosclerosis^26–28^. Most animal models with atherosclerosis have demonstrated augmented expression of eNOS in atherosclerotic arteries, despite the presence of endothelial dysfunction^27,28^. Ozaki et al. suggested that atherosclerosis accelerated by the overexpression of eNOS in apoE-deficient mice was caused by ROS from the endothelium through eNOS dysfunction^26^. In specimens from human coronary atherectomy, eNOS gene expression was higher in patients with acute coronary syndromes than in patients with stable angina^29^. These reports suggested that eNOS function rather than eNOS protein expression is important for maintaining or increasing NO bioactivity.

Several pharmacological studies have reported associations between eNOS activity and BH4 availability. BH4 is a crucial cofactor for a set of metabolically important enzymes, including NOS. Shinozaki et al. reported that deficiency of BH4 contributes to endothelial dysfunction through reduced activity of eNOS and increased ROS generation in insulin-resistant rats^30^. Excess ROS impairs endothelial cells and further limits the biologic activity of eNOS by reacting with NO^30^.

In our study, rat models of CBI demonstrated overexpression of both DHFR and eNOS in the bladder. DHFR can regenerate BH4 from BH2 in the salvage pathway of BH4 synthesis^31^. On the other hand, expressions of SR and GCH-1, which are associated with BH4 synthesis via the de novo pathway, did not differ significantly between the CBI and Control groups. These results suggested that endothelial dysfunction induced overexpression of eNOS to prevent progression of atherosclerosis by NO production in the CBI rat model. To ensure continuous eNOS function, endothelial dysfunction also increased expression of DHFR to produce more BH4 via the salvage pathway in the rat model. We consider that the marked arterial occlusive disease with wall thickening in the bladder arterioles of the CBI group meant that the salvage pathway could not supply sufficient BH4 relative to levels of eNOS protein, despite the increased DHFR expression in the bladder arteriolar endothelium of the rat model.

This study showed that chronic BH4 supplementation suppressed arterial wall thickening of the common iliac arteries and bladder arterioles. We selected a daily oral BH4 dose of 10 mg/kg based on the dose used by Shinozaki et al.^30^ In our rat model, chronic oral administration with this dose of BH4 was able to prevent BH4 deficiency despite eNOS overexpression, resulting in suppression of arterial wall thickening in the bladder. No change in expression of DHFR was evident in the CBI/BH4 group compared with the Control group, which may also indicate that rats in the CBI/BH4 group were given sufficient BH4 relative to the levels of eNOS protein. We consider that chronic BH4 supplementation suppressed arterial wall thickening of the common iliac arteries by the same mechanisms at work in bladder arterioles. Recently, attention has focused on a possible role of BH4 availability in regulating NO-mediated endothelial function. Oral administration of BH4 to insulin-resistant rats was demonstrated to restore endothelium-dependent vasodilation and relieve vascular oxidative stress, at least in part through eNOS activation^30^. In hypercholesterolemic apoE-deficient mice, ingesting BH4 in drinking water slowed the progression of atherosclerosis^32^. Administration of BH4 improves some features of endothelial dysfunction in smokers and in patients with hypercholesterolemia^20,33,34^. Increased HIF1-α expression is well known to reflect conditions of ROS overproduction. In this study, HIF1-α was decreased in the CBI/BH4 group as compared with the CBI group. The decreased HIF1-α may mean that BH4 availability protects against endothelial dysfunction and maintains bladder blood flow by suppressing arterial wall thickening with neointimal formation in rats. In the CBI/BH4 group, significant improvements in muscle strip contractility, cystometric parameters and percentage collagen in the muscle layer were seen compared with the CBI group. BH4 supplementation could protect against bladder contractility and morphology by avoiding chronic ischemia, thus preventing bladder hyperactivity in rats.

We consider that BH4 administration is safe. In this study, rats in the CBI/BH4 group did not show any decrease in body weight. In addition, long-term oral administration of BH4 reportedly shows no adverse effects in hypercholesterolemic patients and healthy volunteers^34^. BH4 was approved for human use in Japan in 1992 for the treatment of hyperphenylalaninemia. Oral administration of BH4 may represent a new pharmacotherapy to prevent atherosclerosis and chronic ischemia-related LUTD in humans. In addition, oral administration of BH4 may prevent coronary artery disease, cerebral infarction and renal dysfunction, as diseases caused by atherosclerosis.

Several limitations to this study must be considered when interpreting the results. We did not measure actual bladder blood flow. However, we believed that increased HIF-1α expression in the bladder and increased percentage of collagen in the muscle layer reflected chronic ischemia^6–8^. We also did not measure plasma concentrations of BH4. However, we considered oral administration of BH4 was appropriate, because many studies have demonstrated that oral supplementation could affect endothelial dysfunction^30,32^. In our study, we did not examine the effect of BH4 in control rats. Several studies reported that BH4 did not affect NO production from aortic vessels with endothelium and myocardium in normal rats^30,35^. They also demonstrated that BH4 did not affect blood pressure and cardiac function. So we consider that oral administration of BH4 do not affect NO production and lower urinary tract function in normal rats. This study did not evaluate the relationship between contractions of muscle strips and NO. As NO can affect contractile activity of the urothelium and lamina propria^36^, changes in NO biosynthesis may contribute to decreased muscle strip contractility in the CBI group.

Our results suggest that arterial occlusive disease increases eNOS and DHFR in the bladder of rats to protect lower urinary tract function by promoting NO bioavailability via the salvage pathway of BH4 synthesis. However, DHFR could not synthesize sufficient BH4 relative to the increased eNOS in the CBI rat model, resulting in chronic ischemia-related LUTD. Chronic treatment with BH4 may prevent LUTD by avoiding increases in ROS and neointimal formation through the maintenance of NO bioavailability. One implication of this study was that BH4 may be therapeutically useful in preventing atherosclerosis and chronic ischemia-related LUTD.

## Materials and methods

This experimental protocol complied with set guidelines for animal experiments. The protocol was reviewed and approved by the Animal Ethics Committee at Fukushima Medical University (#2019015).

### Experimental design

Adult Sprague–Dawley male rats (16 weeks old) were divided into Control, CBI and CBI/BH4 groups. The Control group received regular diet for 8 weeks (n = 10). The CBI group received balloon endothelial injury of bilateral iliac arteries, and was given a 2% cholesterol diet for 8 weeks after the procedure to induce arterial occlusion-related CBI (n = 10). The CBI/BH4 group received the same procedure and diet as the CBI group. In addition, rats in the CBI/BH4 group were orally administered BH4 at 10 mg/kg/day once daily using oral zondes for 8 weeks (n = 10). The BH4, which was generously donated by Daiichi Sankyo (Tokyo, Japan), was dissolved in saline (3 ml). In the Control and CBI groups, vehicle (saline) alone was administered in the same manner. After cystometrogram (CMG) recording in conscious animals, rats from each group were euthanized, and the bladders and common iliac arteries were harvested for pharmacological and histological examinations. We used Western blotting to measure expression levels of eNOS, SR, GCH-1, DHFR and the oxidative stress marker HIF1α in bladder tissue.

### Iliac artery endothelial injury

A 2-Fr Fogarty arterial embolectomy catheter was passed through the femoral artery into the common iliac artery under anesthesia with 2% isoflurane. The balloon was inflated with air, then withdrawn from the common iliac artery to the femoral artery ten times on each side to create endothelial injury in the common and external iliac arteries^6,7^.

### Cystometry in conscious, freely moving rats

The bladder dome was delivered outside the rat body and a small incision was made under anesthesia with 2% isoflurane. The polyethylene catheter with a cuff was inserted through the incision and anchored with a 7-zero polypropylene suture. The proximal catheter was tunneled subcutaneously to exit at the nape of the neck^6–8^. Cystometrogram recordings were performed in conscious, freely moving rats 3 days after catheter implantation^6–8^. The bladder catheter was connected to a pressure transducer and microinjection pump. Room-temperature saline was infused into the bladder at a rate of 10 ml/h. The pressure transducer was connected to an AP-601G transducer amplifier (Nihon Kohden, Tokyo, Japan) and subsequently connected to a MacLab 4/20 data acquisition board. An electric balance with urine collection system was placed under the metabolic cage and connected to the MacLab 4/20 data acquisition board. Rats were given at least 30 min for voiding patterns to stabilize. Reproducible micturition cycles were recorded for 60–90 min. The cystometric parameters investigated were micturition interval (MI), bladder capacity (Bcap), mean voided volume (VV), residual volume (RV), basal pressure (BP), threshold pressure (TP), maximum pressure (MP) and bladder compliance.

### Organ bath study

Rats were sacrificed after cystometrogram recording, and bladders were harvested and weighed. Two or three strips with mucosa (approximately 5 × 2 mm) were cut longitudinally from the middle part of the posterior wall of the bladder. These strips were suspended in a 25-ml organ bath containing Krebs solution. Strip tensions were measured isometrically with a TB-621 force displacement transducer (Nihon Kohden). Each strip was subjected to 1 g of resting tension and allowed to stabilize for 60 min. Contractions were recorded as changes in tension from baseline in response to KCl (80 mM), electrical field stimulation (EFS) (1, 2, 4, 8, 16 and 32 Hz for 10 s with 0.5-ms pulses at 50 V and 2 min of delay between train pulses), 1 mM ATP and carbachol (concentration from 1 nM to 1 mM). All drugs were obtained from Wako Pure Chemical Industries (Tokyo, Japan)^7^.

### Histological examination

The common iliac arteries and bladders from each group were fixed in 10% neutral-buffered formalin, embedded in paraffin and cut into 5-μm sections. Slides were cleared with xylene, dehydrated and used for EM staining. Wall thickness of the common iliac artery was determined by averaging wall thickness at four distinct locations in each sample. Computer-assisted histomorphometric analysis of the EM-stained bladder tissues was performed using a microscope and cellSens Dimension image analysis software (OLYMPUS, Tokyo, Japan)^7^. The percentage of collagen in the bladder muscle layer was based on area calculations of connective tissue (blue-stained) and smooth muscle (red-stained) in four randomly selected high-power fields from each rat. The percentage of collagen in the muscle layer was calculated for every high-power field as the sum of blue-stained areas divided by the sum of all red- and blue-stained areas.

For immunohistochemical examinations of bladder tissue, sections were deparaffinized, and endogenous peroxidase was quenched with 0.3% H_2_O_2_. Nonspecific immunoglobulin G binding was blocked with 5% skim milk. Sections were incubated overnight at 4 °C with primary antibodies for eNOS (GR 32318642, 1:200; Abcam, Cambridge, United Kingdom). After rinsing three times in phosphate-buffered saline for 5 min, appropriate species-directed secondary antibodies (Signal Stain Boast IHC Detection Reagent; Cell Signaling Technology, Danvers, USA) were applied to the sections at room temperature. When substances staining positive were located in the cytoplasm as dark brown granules, these cells were judged as positive on the immunohistochemical evaluation.

### Protein extraction, sodium dodecyl sulfate (SDS)-polyacrylamide gel electrophoresis and Western blotting

The bladders were frozen with liquid nitrogen. Tissues were homogenized, and proteins were extracted with urea (8 M) and dithiothreitol (10 mM). Total protein concentrations of samples were measured with NanoDrop Lite UV–Vis Spectrophotometer (Thermo Fisher Scientific, Waltham, MA). Samples were then mixed with 5 × SDS sample buffer and boiled (4 min). Each sample, containing 10 μg of total protein, was loaded onto an acrylamide gel, and proteins were separated by electrophoresis and blotted onto a PVDF membrane. Membranes were blocked for 1 h with 1% polyvinylpyrrolidone in TBS-T buffer (20 mM Tris pH 7.5, 0.5 M NaCl, 0.1% Tween 20). After washing with TBS-T, membranes were kept at 4 °C overnight with the primary antibody (1:1000 dilution) including the rabbit monoclonal antibodies anti-hypoxia inducible factor-1α (HIF-1α) (#I1212; Santa Cruz, Dallas, TX), anti-GCH-1 (GR 781669), anti-SR (GR 380677), anti-DHFR (GR 781669), and anti-eNOS (GR 32318642) (all from Abcam), and the mouse monoclonal antibody anti-β actin (#059M4770V, 1:3000; Sigma-Aldrich, St Louis, MO). Membranes were then washed four times with TBS-T and incubated for 50 min with horseradish peroxidase-conjugated second antibodies (1:1000 dilution; Promega, Madison, WI). All antibodies were diluted with Can Get Signal Immunostain (Toyobo Company, Osaka, Japan). Following three washings with TBS-T, bands were visualized using SuperSignal West Dura Extended Duration Substrate (Thermo Fisher Scientific), and imaged using a ChemiDoc XRS plus system (BIO-RAD, Hercules, CA)^8^. We washed the membranes with WB stripping Solution Strong to use the same membranes twice (Nacalai Tesque, Kyoto, Japan). With the first wash, anti-HIF-1α, anti-GCH-1, anti-SR, anti-DHFR or anti-eNOS was used. With the second wash, anti-β actin was used as a loading control.

### Statistical analysis

Continuous variables were statistically analyzed using one-way analysis of variance with Tukey’s test to investigate differences among the three groups. Data were analyzed using IBM SPSS Statistics version 21 software (Statistical Package for the Social Sciences, Chicago, IL). Values of *p* < 0.05 were considered significant. GraphPad Prism 5 (GraphPad Software**,** San Diego, CA) was used to calculate EC_50_.
